# Supplementary material for: Balancing efficiency and clinical quality in dialysis centers: Insights from a nationwide DEA study in Taiwan
Source: PLoS One. 2026 Feb 12;21(2):e0343125. doi: 10.1371/journal.pone.0343125 (PMC12900352; doi:10.1371/journal.pone.0343125)
Supplement: Attachment — Submitted filename: Plos review_11 Nov 25.docx [file pone.0343125.s001.docx]

Title of the study: Balancing Efficiency and Clinical Quality in Dialysis Centers: Insights from a Nationwide DEA Study in Taiwan

Reviewed by: Maya Annie Elias

Date: 11-11-25

Dear authors,

I have carefully reviewed the manuscript and would like to congratulate you on undertaking this important study. Managing and analysing such a large dataset is a challenging task. My detailed comments and suggestions are provided below.

1. Conceptual clarity: Clinical quality vs. clinical effectiveness

While the study title is “Balancing Efficiency and Clinical Quality in Dialysis Centers,” the objective mentioned in the abstract, as well as in the main text, states that the study is to *examine the relationship between operational efficiency and clinical effectiveness*. Subsequently, in the methods section, the term clinical quality reappears. Although these concepts are related, they are not interchangeable and are measured differently. Based on the variables and analyses presented, it appears that the study primarily examines clinical quality rather than clinical effectiveness. I recommend using clinical quality consistently throughout the manuscript and ensuring conceptual alignment across the title, objectives, and methods.

2. Introduction of the DEA approach: The Data Envelopment Analysis (DEA) approach should be introduced and briefly explained either at the end of the Introduction section or as part of the method section. Since DEA is a relatively specialised method, readers may not be familiar with it. Please describe the rationale for using DEA, the model applied and how it is suited to assess efficiency in dialysis centres. Currently, the limited information provided under the subheading *efficiency score* is unclear.
3. Clarification of unit of analysis and variable selection: In Line 115, you indicate that the unit of analysis is the dialysis centre, yet the variables described are predominantly individual-level (patient-level) indicators. If the analysis is indeed at the facility level, it would be important to include facility-level parameters that reflect structural and process aspects of care quality- such as cleanliness, infection control measures, water quality assurance, and availability of support services.
4. Please clarify how variables under efficiency and quality were conceptualised and developed. Were these guided by any national or international quality frameworks or standards for dialysis centres? If so, please cite and describe these sources in the Methods section.
5. The operational definition of mortality (Line 121) is not clear. Please revise that.
6. Need for descriptive overview before analysis: Before the univariate and correlation analyses, it would strengthen the paper to provide a descriptive summary of key variables, including key characteristics of dialysis centres, patient profiles, and outcomes. This would help readers, especially policymakers and practitioners, to better understand the functioning and variability among dialysis centres in Taiwan. Currently, the results focus mainly on statistical correlations, which yield limited new insights without context. A more structured descriptive comparison across different types of dialysis centres could enhance the originality and policy relevance of the findings.
7. Discussion: The Discussion section should be more closely aligned with the findings presented. At present, it appears somewhat speculative, for example, lines 215–221, where efficiency is discussed without sufficient grounding in the results. As noted earlier, including a descriptive overview of variables in the Results section would help substantiate these interpretations. Similarly, in Lines 224–236, you discuss about chain-affiliated centres; however, the cost factor is not included in the results. A comparison of the cost of dialysis sessions across different types of centres would have added a valuable perspective to this discussion, particularly regarding accessibility and equity of care.
8. Please also include a “Strengths and Limitations” subsection following the Discussion. In lines 213 and 214, you mention that *variables such as patient education, dialysis duration, and comorbidities, which significantly affect outcomes, were unavailable*. Since these are very important components of quality of care, not including those in your analysis is an inherent limitation of the study- these should be explicitly acknowledged in the manuscript.
