# Supplementary material for: Balancing efficiency and clinical quality in dialysis centers: Insights from a nationwide DEA study in Taiwan
Source: PLoS One. 2026 Feb 12;21(2):e0343125. doi: 10.1371/journal.pone.0343125 (PMC12900352; doi:10.1371/journal.pone.0343125)
Supplement: Attachment — Submitted filename: 1_Response to Reviewers_final.docx [file pone.0343125.s002.docx]

Reviewer #1: Dear authors,

Your manuscript presents a relevant and timely investigation into the interplay between operational efficiency and clinical quality in dialysis centers. The use of Data Envelopment Analysis (DEA) applied to a nationally representative dataset in Taiwan contributes meaningful insights to the health systems literature—particularly in the context of resource-constrained environments. The study is well-structured, and the narrative is clearly articulated.

The findings have potential implications for healthcare policy, quality management, and the economic evaluation of chronic disease care services. However, certain methodological and analytical aspects warrant further clarification or enhancement to strengthen the robustness and applicability of the study's conclusions.

Below is a detailed assessment based on key elements of research design and scientific merit.

(1) Study Design and Level of Evidence

Your study is a retrospective cross-sectional analysis based on aggregated facility-level data. In the hierarchy of evidence, this design provides hypothesis-generating insights but does not support causal inference.

Although DEA is a well-established method for efficiency analysis, its results are sensitive to model specification, input-output selection, and contextual constraints. These limitations are partially acknowledged but would benefit from further discussion.

Recommendation:

Please emphasize in the limitations section that, due to its cross-sectional and ecological nature, the study does not allow for causal conclusions regarding efficiency and clinical outcomes.

Author’s Response:
I appreciate your comments and have strengthened the limitation section to explicitly note that the study design does not permit causal conclusions regarding the relationship between operational efficiency and clinical outcomes.

Lines: 374-380

Limitations and Strengths

“This study has several limitations, but also notable strengths. First, its cross-sectional and ecological design limits the ability to draw causal inferences between efficiency indicators and clinical outcomes. Because the analyses were conducted using aggregated, facility-level data rather than individual-level information, the observed associations may not accurately reflect relationships that occur among individual patients. This reliance on group-level measures also introduces the potential for ecological fallacy, and the cross-sectional nature of the data prevents establishing temporal ordering, making causal interpretations inappropriate.”

(2) Clarity of Research Question and Objectives

The research objective is clearly defined and clinically relevant. You address a critical question: How do organizational structures and ownership models influence the balance between operational efficiency and clinical quality in dialysis care?

This question is particularly pertinent in aging populations and settings with universal health coverage.

Strengths:

Well-formulated aim.

Clear alignment with global health policy discussions.

Addresses a current gap in the efficiency-effectiveness literature.

Author’s Response:

Thank you for your positive comments and supportive feedback.

(3) Methodology and Internal Validity

You employed a DEA model with variable returns to scale (VRS/BCC) to estimate technical efficiency, followed by multiple regression analyses. The methodology is generally sound but requires additional details and refinement.

Points for consideration:

Selection Criteria: The manuscript does not fully explain the inclusion/exclusion criteria for the 578 centers. Was this the entire population or a subset?

DEA Model Justification: While VRS is appropriate, it would be helpful to provide a rationale for the specific input and output variables chosen, including any theoretical or empirical justification.

Input-Output Correlation: Have you tested for multicollinearity among input/output variables? This is particularly important in DEA, where redundancy can distort the frontier.

Recommendation:

Include a more detailed description of the DEA specification process, including variable selection criteria, orientation (input vs. output), and possible sensitivity analyses.

Author’s Response:

Lines 121-126: We have revised the population and the inclusion and exclusion criteria as follows:

“Because hemodialysis is the predominant dialysis treatment in Taiwan, this study focused exclusively on hemodialysis (HD) data, which included all 578 HD centers nationwide. These centers represent the entire population of hemodialysis facilities, whereas peritoneal dialysis centers were excluded from the analysis. The unit of analysis was the individual dialysis center, and no identifiable patient-level or detailed organizational information was available to the researchers.”

Lines 232-237: We have revised the rationale for the input and output variables, as well as our investigation of multicollinearity, as follows:

“…For dialysis centers, the selected input variables included the number of beds, number of nephrologists, number of nurses, number of dialysis technicians, number of other clinical staff full-time equivalents (FTEs), and the number of patients occupying a dialysis station per day. These inputs were chosen because they are closely related to care quality and have been supported in previous studies, indicating their suitability for inclusion in our model [3, 17]. The output variable was the number of dialysis patients.”

17. Donabedian A. (2005). Evaluating the quality of medical care. 1966. *The Milbank quarterly, 83*(4), 691–729.

Lines 208-211; 242-249: We thank you for this helpful suggestion on sensitivity test. We conducted sensitivity analyses by excluding dialysis centers with extreme scale characteristics, defined as centers in the top and bottom 5% of total patient visits and those in the top and bottom 5% of number of beds. Multiple regression analyses were re-estimated for all seven dependent variables. The results were largely consistent with those from the full sample. The direction and magnitude of the estimated associations remained stable across all outcomes. One exception was the model with Kt/V as the dependent variable when excluding centers with extreme numbers of beds, in which the association became statistically non-significant, although the estimated coefficient remained similar in magnitude and direction. Importantly, the overall pattern of results and the study’s conclusions were unchanged, indicating that the findings are not driven by dialysis centers with extreme patient volume or capacity.

We revised the text for methods and results.

In the section of Methods, we revised it as follows (Lines 200-203).

“Finally, to assess the robustness of the findings, sensitivity analyses were performed by excluding dialysis centers with extreme scale characteristics. Specifically, centers in the top and bottom 5% of total patient visits and those in the top and bottom 5% of number of beds were excluded. Multiple regression analyses were then re-run for all seven clinical outcome variables.”

In the section of results (Lines 252-259)

“Results of Sensitivity Analyses

Overall, the sensitivity analyses produced results that were consistent with the primary analyses. The direction and magnitude of the associations between operational efficiency, clinical outcomes, and organizational characteristics remained largely unchanged across all seven dependent variables. In the model with Kt/V as the dependent variable, exclusion of centers with extreme numbers of beds resulted in a loss of statistical significance, although the estimated effect size and direction were similar to those observed in the full-sample analysis. These findings suggest that the main conclusions are robust and not driven by dialysis centers with extreme patient volume or bed capacity.”

We also provided two supplementary Tables presenting the results of the sensitivity analyses, which are included at the end of the response document.

Lines 238-250 (in the section of Results)

“Multicollinearity was assessed using variance inflation factors (VIF), tolerance statistics, and condition index diagnostics, following established methodological guidelines [23-25]. All VIF values were below commonly accepted thresholds (VIF < 5, or under more liberal criteria, < 10), and all tolerance values exceeded 0.20, indicating no evidence of substantial pairwise multicollinearity.

Condition index diagnostics were further evaluated in conjunction with variance-decomposition proportions [23]. Although one condition index exceeded 30 in the model including the for-profit indicator, only a single variable (percentage of female patients) exhibited a high variance-decomposition proportion (0.96) for that dimension, while no other variables showed substantial variance proportions. According to established criteria, problematic multicollinearity is indicated only when two or more variables exhibit large variance proportions associated with the same high condition index. Therefore, these diagnostics do not suggest the presence of meaningful multicollinearity in the model. The VIF, tolerance, and condition index values have been added to Table 3.”

23. Belsley, D. A., Kuh, E., & Welsch, R. E. (1980). Regression diagnostics: Identifying influential data and sources of collinearity. New York: Wiley.

24. Kutner, M. H., Nachtsheim, C. J., Neter, J., & Li, W. (2005). Applied linear statistical models (5th ed.). McGraw-Hill.

25. Menard, S. (1995). Applied logistic regression analysis. Sage.

(4) Statistical Analysis

Your statistical approach is appropriate for an observational study. The use of Spearman’s correlation and multiple regression models is sound, and the results are clearly presented.

However:

The adjusted R² values are relatively low across models, indicating limited explanatory power.

The risk of omitted variable bias remains, given that patient-level covariates (e.g., duration of dialysis, nutritional status, adherence, SES) are not available.

Recommendation:

Discuss the implications of the low R² values more explicitly and consider exploring alternative modeling strategies or interaction terms in future research.

Author’s Response:

Lines 381-387: In the Limitations section, we have added the following statement to explain the low R² values and suggestion for future improving strategies:

“Second, the model’s R² values ranged from 9% to 10%, and were particularly low for hemoglobin (3.9%) and CTR (3.7%), indicating that only a small proportion of the variance in these outcomes is explained by the included variables. This is not unexpected, as clinical outcomes are influenced by many factors beyond structural and staffing characteristics. Therefore, caution is warranted when interpreting these results. Future research could consider alternative modeling strategies, such as hierarchical models that account for clustering within dialysis centers, or interaction terms to explore how combinations of staffing levels, patient load, and facility size jointly affect outcomes, which may provide more actionable insights.”

(5) Results Interpretation and Discussion

The discussion section is balanced and thoughtful. You correctly highlight the trade-offs between operational efficiency and certain clinical quality indicators (e.g., URR, Kt/V, Ca × P). Your analysis of ownership models and chain affiliation is well-integrated with the existing literature.

Strengths:

Strong comparative analysis between chain-affiliated vs. independent centers.

Inclusion of economic theory perspectives enriches the interpretation.

Citations are current and relevant.

Suggestion for Improvement:
Expand the discussion on policy implications. For example:

How might your findings inform accreditation systems, reimbursement models, or health insurance incentives in Taiwan or elsewhere?

Author’s Response:

Lines 349–360: We revised the “Implications and Future Directions” section as follows:

“Implications and Future Directions

This study offers several implications for policy and practice. Policymakers should consider regulatory frameworks that align efficiency incentives with patient-centered outcomes, ensuring that financial performance does not compromise care quality. In Taiwan, for example, accreditation systems could incorporate minimum quality thresholds—such as URR, Kt/V, and Ca × P control—to ensure that highly efficient centers continue to meet essential clinical standards. Reimbursement models under the National Health Insurance (NHI) could also integrate blended or pay-for-performance approaches that reward not only efficient resource use but also consistently strong clinical outcomes, such as stable hemoglobin levels or improved dialysis adequacy. Additionally, health insurance incentives that promote adherence to evidence-based protocols may help counterbalance potential efficiency–quality trade-offs. Collaboration between independent and chain-affiliated centers could further promote equity in resource distribution, dissemination of standardized protocols, and overall performance improvement across the dialysis sector.

From a systems operations perspective, administrators can apply these findings to refine dialysis center management. Investments in health IT systems, workforce development, and AI-enabled tools can enhance both efficiency and care delivery. Flexible operational strategies, such as modular staffing models and data-informed workflow redesign, may help optimize service capacity without eroding care standards. Future research should integrate multi-level data—linking patient characteristics, organizational structures, and financial parameters—to better understand the causal mechanisms underlying these associations. Longitudinal studies are especially needed to examine how organizational strategies and incentive structures influence clinical outcomes over time. Embracing a systems-thinking approach will be essential to sustaining high-quality, efficient dialysis care in an era of increasing demand and resource constraints.”

(6) Risk of Bias and Conflicts of Interest

You have appropriately declared no competing interests, and the study protocol was reviewed and approved by the National Cheng Kung University Research Ethics Committee. Data anonymity is preserved.

However, the following risks of bias should be discussed more clearly:

Selection bias: Particularly if not all dialysis centers were included.

Ecological bias: Outcomes are attributed at the center level, yet clinical variables may vary significantly at the patient level.

Confounding: Control variables are limited and do not fully account for center-level heterogeneity.

Recommendation:
Include a subsection explicitly addressing these sources of bias and their potential influence on findings.

Author’s Response:

Thank you for your constructive comments and suggestions. Because our study included the complete population of hemodialysis centers, selection bias is unlikely to be a concern. Regarding the issue of ecological bias, we have revised the limitation section accordingly and incorporated additional discussion of potential confounding factors.

Lines 373-380:

“…First, the study’s cross-sectional and ecological design limits the ability to draw causal inferences between efficiency indicators and clinical outcomes. Because the analyses are based on aggregate, facility-level data rather than individual-level information, the observed associations may not accurately reflect relationships that occur among individual patients. This reliance on group-level measures also introduces the potential for ecological fallacy, whereby facility-level patterns cannot be assumed to apply to individuals. Moreover, the cross-sectional nature of the data prevents establishing temporal ordering, making it impossible to determine whether efficiency influences clinical outcomes or the reverse. Therefore, readers should interpret any associations with caution, particularly regarding causality.”…

Lines 388-398:

“Third, although the analyses adjusted for key patient characteristics such as age, gender, and comorbidity burden, other relevant sources of confounding could not be accounted for. Information on medication use, nutritional status, and adherence behaviors—all of which influence albumin and calcium–phosphate control—was unavailable. Additionally, facility-level contextual variables such as geographic characteristics, local market competition, and neighborhood socioeconomic environment were not included. These unmeasured factors may have affected both operational efficiency and clinical outcomes.”

(7) References and Scientific Contribution

Your reference list is comprehensive and well-curated, including seminal works on DEA, dialysis quality measures, and health systems performance.

The study adds value by:

Applying a nationwide dataset in a real-world context.

Linking organizational models to measurable clinical indicators.

Reinforcing the need to integrate efficiency and quality metrics in healthcare evaluations.

Author’s Response:

Thank you for your positive comments and supportive feedback.

(8) Overall Recommendation

Recommendation: Minor Revisions

Your manuscript is suitable for publication after minor revisions that enhance methodological transparency, deepen the discussion on policy implications, and better acknowledge the study's design limitations.

Author’s Response:

We sincerely thank you for the professional and insightful comments. We have carefully addressed all suggestions, including enhancing methodological transparency, strengthening the discussion of policy implications, and more clearly acknowledging the study’s design limitations. We believe these revisions have substantially improved the clarity and rigor of the manuscript, and the current version is much stronger than the previous submission.

Reviewer #2: My detailed comments and suggestions are provided below.

1. Conceptual clarity: Clinical quality vs. clinical effectiveness
While the study title is “Balancing Efficiency and Clinical Quality in Dialysis Centers,” the objective mentioned in the abstract, as well as in the main text, states that the study is to examine the relationship between operational efficiency and clinical effectiveness. Subsequently, in the methods section, the term clinical quality reappears. Although these concepts are related, they are not interchangeable and are measured differently. Based on the variables and analyses presented, it appears that the study primarily examines clinical quality rather than clinical effectiveness. I recommend using clinical quality consistently throughout the manuscript and ensuring conceptual alignment across the title, objectives, and methods.

Author’s Response:
We appreciate your comments and have revised the manuscript accordingly by replacing “clinical effectiveness” with “clinical quality.” Please refer to the updated wording in the Abstract and Introduction sections (lines 56, 90, 99, 150, 273, and 411).

2. Introduction of the DEA approach: The Data Envelopment Analysis (DEA) approach should be introduced and briefly explained either at the end of the Introduction section or as part of the method section. Since DEA is a relatively specialised method, readers may not be familiar with it. Please describe the rationale for using DEA, the model applied and how it is suited to assess efficiency in dialysis centres. Currently, the limited information provided under the subheading efficiency score is unclear.

Author’s Response:

We thank you for the helpful suggestion. We have added a brief introduction to the Data Envelopment Analysis (DEA) approach at the end of the Introduction. The revision now explains the rationale for using DEA, the specific model applied, and why it is appropriate for assessing operational efficiency in dialysis centers. This additional information clarifies the calculation and interpretation of efficiency scores for readers who may be unfamiliar with this methodology.

Lines 92-108:

“Quality of care in dialysis is measured through key indicators such as dialysis adequacy (Kt/V and URR), anemia management (hemoglobin levels), bone metabolism (calcium-phosphate balance), and hemodynamic stability [5]. These clinical markers directly influence patient survival and well-being, underscoring the need for performance benchmarking. To evaluate operational efficiency in dialysis centers, we employed Data Envelopment Analysis (DEA), a non-parametric method used to assess the relative efficiency of decision-making units with multiple inputs and outputs [6]. DEA is particularly suited for healthcare settings because it allows simultaneous consideration of multiple resource inputs (e.g., staff, equipment) and outputs (e.g., clinical quality indicators) without assuming a specific functional form (Zakowska & Godycki-Cwirko, 2020). In this study, we applied the BCC model to calculate efficiency scores for each center, where a score of 1 indicates a fully efficient center relative to its peers (Jia & Yuan, 2017). These efficiency scores provide an objective measure of operational efficiency, which we subsequently related to clinical-quality indicators using regression analyses to identify best practices in dialysis management [6–7]. However, while efficiency improvements streamline operations, excessive focus on cost reduction may compromise individualized patient care [8, 9]. Prior studies suggest that highly efficient hospitals may experience higher mortality rates, highlighting the delicate balance between operational efficiency and patient-centered care [10].”

6. Cook , W. D., & Seiford, L. M. (2009). Data envelopment analysis (DEA) – Thirty years on. *European Journal of Operational Research, 192*(1), 1-17.

7. Zakowska, I., & Godycki-Cwirko, M. (2020). Data envelopment analysis applications in primary health care: a systematic review. *Family Practice, 37*(2), 147–153.

8. Jia, T., & Yuan, H. (2017). The application of DEA (Data Envelopment Analysis) window analysis in the assessment of influence on operational efficiencies after the establishment of branched hospitals. *BMC Health Services Research, 17*(1), 265.

3. Clarification of unit of analysis and variable selection: In Line 115, you indicate that the unit of analysis is the dialysis centre, yet the variables described are predominantly individual-level (patient-level) indicators. If the analysis is indeed at the facility level, it would be important to include facility-level parameters that reflect structural and process aspects of care quality- such as cleanliness, infection control measures, water quality assurance, and availability of support services.
Author’s Response:

Thank you for this important comment. We agree that clarity regarding the unit of analysis and the nature of our variables is essential.

In this study, the unit of analysis is the dialysis center, and all clinical indicators used in the analysis were facility-level aggregated measures routinely reported to the national registry. Although these indicators originate from individual patient records (e.g., URR, Kt/V, albumin), they are summarized at the center level (using mean values or proportions) and therefore represent facility-level outcomes rather than individual-level data. We have revised the Methods section to explicitly emphasize that these are center-level aggregated quality indicators.

Regarding structural and process quality measures (e.g., cleanliness, infection control protocols, water treatment quality, availability of dietitian/social work services), we fully acknowledge their importance. Unfortunately, such structural/process variables are not available in the national dataset used for this study. We have now added text to the Limitations section to note this constraint and recommend that future research incorporate these dimensions to provide a more comprehensive assessment of dialysis center performance.

Lines 156-160

We revised the section of Methods as follows:

… “The unit of analysis in this study was the dialysis center. Although the clinical indicators originate from individual patient records, all measures used in the analysis were aggregated at the facility level and reported as center-level summary statistics (e.g., mean URR, mean Kt/V, proportion meeting hemoglobin targets). Therefore, all variables included in the regression models reflect organizational-level outcomes rather than individual-level data.”

Lines 394-400. We revised the Limitations as follows:

“…Fourth, the national dataset lacked several important structural and process quality indicators at the facility level, including cleanliness and environmental hygiene, infection control practices, water treatment quality assurance, and the availability of multidisciplinary support services such as dietitians and social workers. These components represent essential dimensions of dialysis center quality and may influence both organizational efficiency and clinical performance. The absence of these measures limits the comprehensiveness of our performance assessment. Future studies incorporating these structural and process indicators would provide a more holistic evaluation of dialysis center performance.”

4. Please clarify how variables under efficiency and quality were conceptualised and developed. Were these guided by any national or international quality frameworks or standards for dialysis centres? If so, please cite and describe these sources in the Methods section.

Author’s Response:
Thank you for your suggestion. We have updated the Methods section to explicitly describe the conceptualization and development of these variables, and to cite the relevant national and international guidelines and DEA applications.

Lines 150-160

“The clinical quality variables in our study—such as dialysis adequacy (Kt/V and URR), hemoglobin levels for anemia management, calcium–phosphate balance, and other biochemical markers—are grounded in widely recognized national and international quality frameworks for dialysis care. These indicators are emphasized in the Kidney Disease Outcomes Quality Initiative (KDOQI) and associated quality measure initiatives, which outline core measures for dialysis adequacy and management of anemia and mineral metabolism that are linked to patient outcomes and survival (Weiner et al., 2024).

Lines 162-181

Efficiency score: We used the Data Envelopment Analysis (DEA) technique to measure the relative technical efficiency scores of each dialysis center. Each dialysis center is called Decision Making Units (DMUs) in DEA. This study used a variable return to scale (VRS, BCC) DEA model in examining the technical efficiency of dialysis facilities, assuming that the average productivity at the most productive scale size may not be attainable for the other scale sizes at which a given dialysis center may be operating [11]. DEA provided efficiency scores by taking account of both multiple inputs and outputs [7, 12]. For dialysis centers, the selected input variables included the number of beds, number of nephrologists, number of nurses, number of dialysis technicians, number of other clinical staff full-time equivalents (FTEs), and the number of patients occupying a dialysis station per day. These inputs were chosen because they are closely related to care quality and have been supported in previous studies, indicating their suitability for inclusion in our model [3, 16] (Donabedian, 2005). The output variable was the number of dialysis patients.

Lines 244-256

“Multicollinearity was assessed using variance inflation factors (VIF), tolerance statistics, and condition index diagnostics, following established methodological guidelines [22-24](Belsley, Kuh, & Welsch, 1980; Kutner et al., 2005; Menard, 1995). All VIF values were below commonly accepted thresholds (VIF < 5, or under more liberal criteria, < 10), and all tolerance values exceeded 0.20, indicating no evidence of substantial pairwise multicollinearity.

Condition index diagnostics were further evaluated in conjunction with variance-decomposition proportions [22]. Although one condition index exceeded 30 in the model including the for-profit indicator, only a single variable (percentage of female patients) exhibited a high variance-decomposition proportion (0.96) for that dimension, while no other variables showed substantial variance proportions. According to established criteria, problematic multicollinearity is indicated only when two or more variables exhibit large variance proportions associated with the same high condition index. Therefore, these diagnostics do not suggest the presence of meaningful multicollinearity in the model. The VIF, tolerance, and condition index values have been added to Table 3.”

14. Weiner, D. E., Delgado, C., Flythe, J. E., Forfang, D. L., Manley, T., McGonigal, L. J., McNamara, E., Murphy, H., Roach, J. L., Watnick, S. G., Weinhandl, E., Willis, K., Berns, J. S., & KDOQI Patient-Centered Quality Measures for Dialysis Care Workshop Participants (2024). Patient-Centered Quality Measures for Dialysis Care: A Report of a Kidney Disease Outcomes Quality Initiative (KDOQI) Scientific Workshop Sponsored by the National Kidney Foundation. American journal of kidney diseases : the official journal of the National Kidney Foundation, 83(5), 636–647.

16. Donabedian A. (2005). Evaluating the quality of medical care. 1966. The Milbank quarterly, 83(4), 691–729.

22. Belsley, D. A., Kuh, E., & Welsch, R. E. (1980). Regression diagnostics: Identifying influential data and sources of collinearity. New York: Wiley.

23. Kutner, M. H., Nachtsheim, C. J., Neter, J., & Li, W. (2005). Applied linear statistical models (5th ed.). Irwin, New York: McGraw-Hill.

24. Menard, S. (2011). Applied logistic regression analysis. Thousand Oaks: Sage Publications, Inc.

5. The operational definition of mortality (Line 121) is not clear. Please revise that.

Author’s Response:

We revised the definition of mortality as follows.

Lines 137 to 141

“Annual mortality for each dialysis center was calculated as the number of deaths divided by total patient-years at risk, with each patient contributing proportionally to the time they received hemodialysis during the year, and expressed as a percentage.”

6. Need for descriptive overview before analysis: Before the univariate and correlation analyses, it would strengthen the paper to provide a descriptive summary of key variables, including key characteristics of dialysis centres, patient profiles, and outcomes. This would help readers, especially policymakers and practitioners, to better understand the functioning and variability among dialysis centres in Taiwan. Currently, the results focus mainly on statistical correlations, which yield limited new insights without context. A more structured descriptive comparison across different types of dialysis centres could enhance the originality and policy relevance of the findings.

Author’s Response:

We thank the reviewer for this valuable suggestion. In the original manuscript, we included a descriptive overview of the 578 dialysis centers, reporting chain affiliation, ownership type, average patient age, comorbidity burden, and efficiency scores (Table 1). To further address the reviewer’s comment, we have now expanded the descriptive section to include additional facility-level clinical outcomes. These enhanced descriptive statistics provide important context for understanding the variability among dialysis centers prior to the correlation and regression analyses.

Lines 216-224:

“Sample Characteristics

The study included 578 hemodialysis centers in Taiwan. Among these, 250 centers (43.3%) were chain-affiliated and 328 (56.7%) operated independently. The majority of centers (426, 73.7%) were for-profit, while 152 (26.3%) were non-profit. On average, patients treated at these centers were 65.5 years old with 2.83 comorbidities, and 48% were female. The mean efficiency score across centers was 0.95 (range: 0.32–1.00). Facility-level clinical outcomes indicated a mean mortality rate of 0.06 (SD = 0.04), mean URR of 0.75 (SD = 0.02), mean Kt/V of 1.41 (SD = 0.09), mean albumin of 3.88 g/dL (SD = 0.18), mean hemoglobin of 10.41 g/dL (SD = 0.59), mean Ca × P of 45.12 (SD = 4.01), and mean cardiothoracic ratio of 0.51 (SD = 0.03). A detailed breakdown of these characteristics is presented in Table 1.”

7. Discussion: The Discussion section should be more closely aligned with the findings presented. At present, it appears somewhat speculative, for example, lines 215–221, where efficiency is discussed without sufficient grounding in the results. As noted earlier, including a descriptive overview of variables in the Results section would help substantiate these interpretations. Similarly, in Lines 224–236, you discuss about chain-affiliated centres; however, the cost factor is not included in the results. A comparison of the cost of dialysis sessions across different types of centres would have added a valuable perspective to this discussion, particularly regarding accessibility and equity of care.

Author’s Response:

We thank you for the valuable suggestions regarding the discussion of efficiency, ownership, and organizational characteristics. In response, we have rewritten the Discussion section to integrate these points more clearly. The revised section now: (1) Explains the observed negative association between efficiency scores and dialysis adequacy (URR and Kt/V) in the context of clinical guideline targets; (2) Discusses ownership effects, highlighting differences between for-profit and nonprofit centers, and incorporates relevant literature [18,23–27] to contextualize mixed findings in prior studies; (3) Addresses the potential role of chain and investor-owned centers in maintaining dialysis adequacy through care pathways, staff training, and monitoring systems; and (4) Emphasizes the importance of balancing operational efficiency with clinical quality and provides recommendations for policymakers and center managers. We believe these revisions directly address your comments and improve the clarity, coherence, and policy relevance of the discussion.

Lines 266-355

“Discussion

The finding that higher efficiency scores were associated with lower URR and Kt/V values suggests that facilities operating more efficiently may be doing so by reducing treatment time or resource intensity, potentially compromising dialysis adequacy. Both URR and Kt/V are key indicators of solute clearance, with clinical guidelines recommending a URR of at least 65% and a single-pool Kt/V (spKt/V) target of approximately 1.4 per treatment to ensure sufficient urea removal and prevent adverse outcomes. The negative association observed in this study indicates that greater operational efficiency may coincide with reduced clearance performance, raising concerns that cost-saving strategies–such as shorter dialysis sessions, lower staffing ratios, or streamlined processes–might inadvertently limit treatment effectiveness. This interpretation is further supported by the positive association between efficiency and the calcium–phosphate (Ca × P) product, where higher values exceed the recommended threshold of <55 mg²/dL² and indicate suboptimal mineral metabolism management. Elevated Ca × P levels are clinically concerning because they are associated with vascular calcification and increased cardiovascular risk. Taken together, these patterns highlight a potential tension between operational efficiency and clinical quality, underscoring the need for balanced performance strategies that safeguard both throughput and patient outcomes.

Ownership type further influenced clinical performance. For-profit centers demonstrated higher URR and Kt/V values, higher albumin levels, and lower Ca × P compared with nonprofit centers, suggesting that investor-owned facilities may allocate resources toward maintaining dialysis adequacy and nutritional monitoring. However, our dataset did not include information on the cost of dialysis sessions across ownership types. Including cost data—such as per-session charges, out-of-pocket expenses, or reimbursement structures—would have provided a more complete perspective on how financial models affect accessibility, equity, and quality. Existing literature underscores the heterogeneous effects of ownership on dialysis care. Lin et al. reported that nephrologist ownership was associated with lower ESA use without worsening anemia control [23], whereas Thamer et al. found that for-profit dialysis facilities administered higher ESA doses than nonprofit facilities, consistent with reimbursement-driven incentives rather than clinical need [26]. Similarly, Eggleston et al. showed that for-profit hospitals respond to financial incentives with variable quality outcomes [24]. While some studies suggest quality advantages for nonprofit hospitals [25], meta-analyses have linked for-profit ownership to higher mortality in certain settings [18,26], and European evidence shows no consistent benefits from for-profit expansion [27]. Overall, these mixed findings highlight the need for context-sensitive interpretation of profit-oriented ownership models in dialysis care.

Chain affiliation and investor ownership may also confer operational advantages. These centers could invest in standardized care pathways, staff training, or real-time monitoring systems that help preserve dialysis adequacy even while pursuing efficiency gains. Importantly, efficiency was not significantly associated with mortality in our models, indicating that reductions in URR/Kt/V and increases in Ca × P do not necessarily translate into measurable short-term mortality differences at the facility level. Nevertheless, these process-oriented deficits–such as lower clearance performance and higher Ca × P–remain clinically meaningful, as prior studies have linked them to higher risks of hospitalization, vascular complications, and long-term morbidity.

Overall, these results underscore the need for policymakers and dialysis center managers to monitor process and biochemical quality indicators, not only cost or throughput metrics, when implementing efficiency-focused strategies. Future research should examine specific organizational practices, including treatment duration, staffing mix, and phosphate-management protocols, and incorporate longitudinal patient-level analyses to determine whether the observed trade-offs persist over time and ultimately affect long-term patient outcomes.”

26. Thamer, M., Zhang, Y., Kaufman, J., Cotter, D., Dong, F., & Hernán, M. A. (2007). Dialysis facility ownership and epoetin dosing in patients receiving hemodialysis. *JAMA, 297*(15), 1667–1674.

8. Please also include a “Strengths and Limitations” subsection following the Discussion. In lines 213 and 214, you mention that variables such as patient education, dialysis duration, and comorbidities, which significantly affect outcomes, were unavailable. Since these are very important components of quality of care, not including those in your analysis is an inherent limitation of the study- these should be explicitly acknowledged in the manuscript.

Author’s Response:

We thank you for the suggestion. We have revised the “Limitations” section into a combined “Limitations and Strengths” section, which now clearly presents both the study’s limitations and its strengths, including the system-level evaluation of dialysis center efficiency, use of DEA and regression modeling, and comparisons across ownership and chain affiliation.

Lines 381-416

“Limitations and Strengths

This study has several limitations, but also notable strengths. First, its cross-sectional and ecological design limits the ability to draw causal inferences between efficiency indicators and clinical outcomes. Because the analyses were conducted using aggregated, facility-level data rather than individual-level information, the observed associations may not accurately reflect relationships that occur among individual patients. This reliance on group-level measures also introduces the potential for ecological fallacy, and the cross-sectional nature of the data prevents establishing temporal ordering, making causal interpretations inappropriate.

Second, the model’s explanatory power was modest. The R² values generally ranged from 9% to 10% and were particularly low for hemoglobin (3.9%) and cardiothoracic ratio (3.7%), indicating that only a small proportion of the variance in these outcomes is explained by the included variables. As clinical outcomes are shaped by multiple patient-, provider-, and system-level factors, these low R² values are not unexpected; however, they underscore the need for caution in interpreting the findings. Future research may benefit from alternative analytic approaches—such as hierarchical modeling or interaction terms—to capture more complex relationships among patient load, staffing patterns, and facility size.

Third, although the analyses adjusted for key patient characteristics such as age, gender, and comorbidity burden, other relevant sources of confounding could not be accounted for. Information on medication use, nutritional status, and adherence behaviors—all of which influence albumin and calcium–phosphate control—was unavailable. Additionally, facility-level contextual variables such as geographic characteristics, local market competition, and neighborhood socioeconomic environment were not included. These unmeasured factors may have affected both operational efficiency and clinical outcomes.

Fourth, the national dataset lacked several important structural and process quality indicators at the facility level, including cleanliness and environmental hygiene, infection control practices, water treatment quality assurance, and the availability of multidisciplinary support services such as dietitians and social workers. These components represent essential dimensions of dialysis center quality and may influence both organizational efficiency and clinical performance. The absence of these measures limits the comprehensiveness of our performance assessment. Future studies incorporating these structural and process indicators would provide a more holistic evaluation of dialysis center performance.

Despite these limitations, the study has several important strengths. Its primary strength lies in its comprehensive, system-level analysis of dialysis center efficiency, incorporating both operational performance and care quality dimensions. By employing Data Envelopment Analysis (DEA) alongside regression modeling, the study provides a rigorous framework to evaluate facility-level efficiency and identify potential trade-offs with clinical outcomes. In addition, comparing independent and chain-affiliated centers, as well as for-profit versus nonprofit ownership, contributes actionable knowledge for system-level improvement strategies and offers valuable insights for policymakers and healthcare managers seeking to optimize resource allocation while maintaining care quality.”

Supplementary Table 1. Sensitivity test 1: Results of Multiple Regression Analyses Excluding Centers with Total Patient Visits in the Top and Bottom 5%

| Variable | Mortality rate | | URR | | KT/V | | Albumin | | Hemoglobin | | Ca × P | | CTR | | Tolerance | VIF | Condition Index |
| --- | --- | --- | --- | --- | --- | --- | --- | --- | --- | --- | --- | --- | --- | --- | --- | --- | --- |
|  | *β* | *SE* | *β* | *SE* | *β* | *SE* | *β* | *SE* | *β* | *SE* | *β* | *SE* | *β* | *SE* |  |  |  |
| Female% | -.012 | .026 | .068*** | .014 | .315*** | .058 | .027 | .112 | -.307 | .369 | 4.498 | 2.486 | .048** | .020 | .924 | 1.082 | 3.089 |
| Age | .003*** | .001 | .000 | .000 | .001 | .002 | -.015*** | .003 | -.029** | .010 | -.446*** | .065 | .001** | .001 | .939 | 1.065 | 5.038 |
| Comorbidity | .006*** | .001 | .000 | .001 | .002 | .002 | .008 | .004 | .037* | .015 | -.002 | .096 | .001 | .001 | .988 | 1.012 | 6.696 |
| Efficiency score | -.019 | .011 | -.014** | .006 | -.056* | .024 | .025 | .047 | -.019 | .160 | 2.681* | 1.031 | -.001 | .008 | .960 | 1.042 | 15.382 |
| Chain | .006 | .004 | .007*** | .002 | .024** | .008 | .022 | .016 | .092 | .055 | -.848* | .358 | .002 | .003 | .834 | 1.199 | 25.000 |
| For-profit | -.004 | .005 | .007** | .002 | .021* | .010 | .073*** | .020 | -.071 | .064 | -1.232** | .431 | -.005 | .003 | .790 | 1.266 | 91.065 |
| *R*^2^ | .106 | | .089 | | .084 | | .093 | | .039 | | .103 | | .039 | |  |  |  |
| *Adjusted R*^2^ | .096 | | .079 | | .073 | | .082 | | .028 | | .093 | | .028 | |  |  |  |
| *F-value* | 10.184*** | | 8.421*** | | 7.887*** | | 8.805*** | | 3.776** | | 9.867**** | | 3.488** | |  |  |  |
| *Note.* *N* = 522; ****p<.001 **p<.01 *p<.05 ;* URR= urea reduction ratio; KT/V= K – dialyzer clearance of urea, T – dialysis time, V – volume of distribution of urea; Ca × P= calcium-phosphate product; CTR= cardiothoracic ratio. | | | | | | | | | | | | | | | | | |

Supplementary Table 2. Sensitivity test 2: Results of Multiple Regression Analyses Excluding Centers with Number of Beds in the Top and Bottom 5%

| Variable | Mortality rate | | URR | | KT/V | | Albumin | | Hemoglobin | | Ca × P | | CTR | | Tolerance | VIF | Condition Index |
| --- | --- | --- | --- | --- | --- | --- | --- | --- | --- | --- | --- | --- | --- | --- | --- | --- | --- |
|  | *β* | *SE* | *β* | *SE* | *β* | *SE* | *β* | *SE* | *β* | *SE* | *β* | *SE* | *β* | *SE* |  |  |  |
| Female% | -.001 | .028 | .068*** | .014 | .321*** | .060 | .060 | .116 | -.307 | .420 | 4.498 | 2.632 | 034 | .020 | .906 | 1.104 | 3.088 |
| Age | .003** | .001 | .000 | .000 | .001 | .002 | -.015*** | .003 | -.028* | .011 | -.463*** | .070 | .002** | .001 | .929 | 1.076 | 5.031 |
| Comorbidity | .005*** | .001 | .000 | .001 | .002 | .002 | .008 | .004 | .044** | .016 | -.006 | .099 | .001 | .001 | .988 | 1.013 | 6.912 |
| Efficiency score | -.011 | .013 | -.015* | .007 | -.053 | .028 | -.032 | .054 | -.112 | .197 | 3.053* | 1.235 | .001 | .010 | .960 | 1.041 | 16.777 |
| Chain | .005 | .004 | .009*** | .002 | .031*** | .009 | .021 | .017 | .075 | .061 | -.844* | .379 | .002 | .003 | .817 | 1.223 | 26.464 |
| For-profit | -.004 | .005 | .008** | .003 | .023 | .011 | .076*** | .021 | -.120 | .075 | -1.336** | .467 | -.004 | .004 | .763 | 1.310 | 93.191 |
| *R*^2^ | .083 | | .098 | | .092 | | .094 | | .038 | | .100 | | .039 | |  |  |  |
| *Adjusted R*^2^ | .072 | | .087 | | .081 | | .083 | | .026 | | .089 | | .028 | |  |  |  |
| *F-value* | 7.415*** | | 8.952*** | | 8.361*** | | 8.525*** | | 3.249** | | 9.104**** | | 3.488** | |  |  |  |
| *Note.* *N* = 500; ****p<.001 **p<.01 *p<.05 ;* URR= urea reduction ratio; KT/V= K – dialyzer clearance of urea, T – dialysis time, V – volume of distribution of urea; Ca × P= calcium-phosphate product; CTR= cardiothoracic ratio. | | | | | | | | | | | | | | | | | |
